# Occupational Allergy to Rat and Mouse in Research Laboratories

**DOI:** 10.1007/s11882-026-01250-z

**Published:** 2026-01-25

**Authors:** Juliette Caron, Anaïs Lemoine, Anne Herman, Florence Libon, Christine Delebarre-Sauvage

**Affiliations:** 1 Allergy unit, Saint Vincent-de-Paul hospital, Boulevard de Belfort, Lille, 59020 France; 2 Catholic University of Lille, Lille, France; 3 Paediatric Nutrition and Gastroenterology Department, Sorbonne Université, APHP- Trousseau Hospital, 26 avenue du Dr Arnold Netter, Paris, 75012 France; 4 Dermatology department, Cliniques Universitaires Saint Luc, Catholic University of Louvain, Brussels, Belgium; 5 Catholic University of Louvain, Ottignies-Louvain-la-Neuve, Belgium; 6 Dermatology department, University Hospital of Liège, Liège, Belgium

**Keywords:** Allergy, Mouse, Rat, Research laboratory, Sensitization, Occupational allergy, Rodent, Review

## Abstract

**Purpose of Review:**

Occupational allergy to rat and mouse in laboratory animal facilities remains underdiagnosed and raises major concerns. This comprehensive review provides an overview of this allergy in literature and recent findings over the last decade.

**Recent Findings:**

The prevalence of rat and mouse allergy among laboratory workers over the past ten years ranges from 4,4% to 30%. Recent studies about sensitization to laboratory rat and mouse focus on rodent epithelial proteins, whereas sensitization to urinary proteins are traditionally studied. Thanks to molecular biology, studies are now paying attention to the allergenic proteins involved in the clinical reaction, notably Rat n 1, Rat n 4, Mus m 1 and Mus m 4.

**Summary:**

Despite known preventive measures, the prevalence of rat and mouse allergy among LAW remains stable over the last decade compared to previous data ranging from 4,4% to 30%. Anaphylaxis remains rare, but surely underdiagnosed. Determination of specific IgE against the different allergenic sources of rats and mice is easy to carry out routinely: epithelial, urinary and blood serum. However, rat and mouse allergenic protein identification remains difficult in clinical routine. The advent of molecular biology, allergen immunotherapy and biologic therapy may perhaps lead to the development of personalized treatments in future.

## Introduction

The Norway rat (Rattus norvegicus) and the house mouse (Mus musculus) are the most frequent animals in laboratory animal facilities (LAF) [1–3]. Widely used in fields such as neuroscience, physiology and toxicology, it is estimated that 111.5 million mice and rats are used in LAF in the United States annually [4]. These rodents, belonging to the superfamily Muroidea, are selected for many reasons: small size facilitating housing, short reproductive cycle, gentle temperament, known genetic background and similarities with human diseases [1]. The Norway rat is often considered the first mammal to have been domesticated for research purposes [5]. The first known documented experiment conducted on these animals was a study of the effects of adrenalectomy published in 1856 in France [6]. Despite the proven usefulness of these rodents in the scientific world, their manipulation is not without risk for workers in LAF. It is well known that laboratory animal workers (LAW), research technicians or researchers, are at risk of sensitization to rats and mice [7–11]. In this context, sensitization is the process by which the immune system develops specific Immunoglobulins E (IgE) to allergenic animal proteins after initial exposure. Exposure to mouse and rat allergens can occur through direct contact or inhalation of aeroallergens from the animal. This literature review aims to summarize research on laboratory rat and mouse allergy over the past 10 years.

## Developing Allergy to Rat and Mouse in Research Laboratory

According to studies published over the last decade, sensitization rates among LAW range from 4,4% to 30% (Table 1). The epidemiological data therefore do not appear to have changed over the past 40 years [12, 13]. Individuals with an atopic background, characterized by a genetic predisposition to develop allergic diseases, are particularly vulnerable to allergens present in the laboratory environment [14]. It appears that LAW with atopic dermatitis are particularly at risk [15]. In Bar Harbor, Maine, United States of America, a single-center study conducted in a research laboratory highlighted that physician-diagnosed eczema was an independent risk factor for incident sensitization to mouse skin prick test (SPT) with a hazard ratio of 5.6 [95% confidence interval (CI), 2.1–15.2]; *p* = 0.001 [15]. Indeed, at 30 months of follow-up, 36% of LAW with eczema versus 14% of those without eczema had developed a positive mouse SPT result (*p* = 0.02).

**Table 1:** Sensitization to rats and mice in research laboratories over the last decade

Reference (year of publication)	Allergens	Exposed population *N*	Sensitized workers *n* (%)
Kang and al. (2021) [7]	Rat	211	29 (13,7%)
	Mouse	213	64 (30%)
Feary and al. (2019) [10]	Rat	743	32 (4,4%)
Simoneti and al. (2017) [8]	Rat and Mouse	453	74 (16,3%)
Yoshimura and al. (2014) [16]	Rat	279	50 (17,9%)
	Mouse	441	62 (14,1%)
Muzembo and al. (2014) [17]	Rat	88	17 (19,3%)
	Mouse	119	23 (19,3%)

Lifelong occupational exposure to animals seems to be associated both with sensitization and symptoms [17]. Moreover, repeated and prolonged contact with rat and mice in sensitized subjects may lead to allergy symptoms [18]. Occupational sensitization to rats and mice is associated with a greater risk of developing symptoms compared to atopic patients without occupational exposure [8]. According to Simoneti et al. [8], LAW have a 2.65-fold higher risk [95% CI, 1.45–4.85] of developing asthma than the atopic population without occupational exposure. They also have a higher risk of developing rhinitis or skin symptoms with a prevalence ratio of 1.25 [95% CI, 1.11–1.40] and 1.36 [95% CI, 1.01–1.85], respectively. A recent Italian multicenter study indicates a 3% sensitization rate for mice and 1% for rat among Italian people with allergic rhinitis or asthma [19].

### Clinical Manifestations of Laboratory Rat and Mouse Allergy

The most common allergic manifestations in LAW in contact with rats and mice are rhinitis, conjunctivitis, asthma and urticaria [7, 8, 20, 21]. Although symptoms are generally mild to moderate, some may be life-threatening [20]. Of 40 cases of animal anaphylaxis induced by bites described in the English-language literature, eight cases of anaphylaxis to laboratory rats and mice are reported (Table 2) [20, 22–27]. This figure is probably an underestimate. According to an online survey sent to institutional officials at laboratory animal facilities in the United States, nine of 198 institutions indicated that 13 LAW had experienced anaphylaxis following a rodent bite [28]. Note that rat and mouse bites do not a priori constitute a risk factor for sensitization [17].

**Table 2:** Published cases of anaphylaxis to laboratory rats and mice induced by bites

	Gender	Age	Animal involved	Anaphylaxis grade*	Skin prick test	Specific IgE	Ref.
1	F	48	Rat	II	Rat = 7 mm	Rat epithelium = 0.99 kUA/L	[20]
2	F	25	Mouse	II	Unavailable	Mouse epithelium = 42.40 kUA/L Rat epithelium = 10.50 kUA/L	[20]
3	M	23	Rat	II	Rat urine = 10 mm Rat dander = 12 mm Rat serum = 16 mm Mouse urine = 12 mm Mouse dander = 5 mm Mouse serum = 16 mm	Rat urine = positive Rat dander = positive Rat serum = positive Mouse urine = positive Mouse dander = positive Mouse serum = positive	[22]
4	M	30	Mouse	II	Mouse urine = 6 mm Mouse dander = 0 mm Mouse serum = 0 mm Rat urine = 3 mm Rat dander = 3 mm Rat serum = 0 mm	Mouse urine = positive Mouse dander = positive Mouse serum = positive Rat urine = positive Rat dander = negative Rat serum = positive	[22]
5	F	35	Rat	IV	Rat urine (1/10000 dilution) = positive	Unavailable	[23]
6	M	50	Rat	III	Unavailable	Unavailable	[24]
7	F	40	Mouse	III	Mouse epithelia > 10 mm Rat epithelia = 10 mm	Mouse urine = 26.00 kUA/L Mouse dander = 17.60 kUA/L Mouse serum = 7.98 kUA/L Rat urine = 24.50 kUA/L Rat dander = 4.15 kUA/L Rat serum = 5.34 kUA/L	[25]
8	M	55	Mouse	III	Mouse epithelium = 6 mm Rat epithelium = 11 mm	Mouse epithelium = 6.17 kU/L Mouse urinary protein = 0.41 kUA/L	[26]

* According to Ring and Messmer grading scale [29]

Prolonged exposure to laboratory animals appears to increase the risk of developing asthma [8, 14, 18]. Both sensitization and exposure contribute independently to decline lung function [8]. Lung function decline is more noticeable in sensitized LAW who continue to be exposed to the animals to which they are sensitized. These data should be confirmed in future, more robust studies. Apart from allergens, exposure to endotoxins also impacts the respiratory status of LAW [30]. This exposure does not appear to be a risk factor for the development of asthma but rather wheezing. For instance, in a cross-sectional study performed in workplaces of two Brazilian universities, 61% of workers exposed to high endotoxin from Gram-negative bacteria concentration reported wheezing in the last 12 months compared to 29% of workers exposed to low endotoxin concentration [30].

## Rat and Mouse Allergenic Proteins

Different allergenic proteins from rats and mice have been characterized (Table 3). They are present in urine, hair, epithelium or dander, milk or blood serum. Lipocalins are major allergens for rats (Rat n 1) and mice (Mus m 1) [31]. They are expressed in almost all vertebrates and seem essential for life as they play roles in spermatogenesis, embryonic development, regulation of oxidative stress and antimicrobial defense [32]. Cross-reactivity between Rat n1 and Mus m1 has been well established [31]. A recent In silico analysis has also pointed out possible crossbet-reactivity between Rat n 1, Mus m1 and other animal lipocalins such as Can f 6 (dog), Equ c 1 (horse) and Fel d 4 (cat) with 62% identity among sequences compared [33]. Recent studies suggest that serum albumin may be the primary driver of cross-sensitization between furry animals (mice, rats, cats, dogs, guinea pigs, rabbits), rather than lipocalins [34, 35].

**Table 3:** Rat and mouse allergenic proteins

Animal	Allergenic proteins	MW (kDa)	Biochemical name	Tissues	Reference
Rat *(Rattus norvegicus)*	Rat n 1 A/ Rat n1B Rat n 4 Rat n 7 Rat n 8 Rat n Gelatin Rat n Phosvitin Rat n Transferrin	16–21	Lipocalin Serum Albumin Immunoglobulin Casein Gelatin Casein Kinase Transferrin	Urine Urine, serum Serum Milk Epithelium Serum	[31, 36–38] [39] [39] [40] [41, 42] [43] [39]
Mouse *(Mus musculus)*	Mus m 1 Mus m 2 Mus m 4 Mus m Gelatin Mus m Muromonab Mus m Phosvitin	17 16 20–23	Lipocalin Unknown Serum Albumin Gelatin Immunoglobulin Casein Kinase	Urine Hair, epithelium Urine, serum Epithelium Serum Urine	[31, 36, 44] [36] [36] [41, 42] [45] [46]

Recent studies about sensitization to laboratory rat and mouse focus on rodent epithelial proteins [7, 10, 47], whereas sensitization to urinary proteins are traditionally studied [10, 15, 37, 48–55]. Few studies on LAW allergy to rat and mouse have focused on protein identification. They only concern urinary proteins: Rat n 1 [53], Rat n 4 [48, 49], Mus m 1 [10, 15, 48], Mus m 4 [48].

## Current Diagnostic Tools in Clinical Routine

### Clinical Relevance

A diagnosis of laboratory animal allergy may be based on history and clinical findings alone [56]. Since rat and mouse allergy can be confused with irritation caused by environmental endotoxins [56] or with an allergy to another laboratory animal, a positive allergy assessment can confirm the diagnosis with certainty. Many studies compare biological data with a symptom questionnaire to diagnose an allergy to laboratory rodent [37, 48, 50, 53, 54]. Allergy assessment is based on the detection of sensitivity to rodent using SPT and/or specific IgE.

### Skin Prick Tests

SPT must be carried out according to the recommendations [57]. Commercial extracts to rodent allergens may be used to perform SPT [25, 26, 58]. But their access is limited in clinical routine. Native rodent hairs can be used if no commercial extract is available [21]. However, for reasons of hygiene, their implementation is questionable. Local allergen extraction is possible but requires a trained research team [58].

### Specific IgE

Determination of specific IgE against the different allergenic sources of rats and mice is easy to carry out routinely : epithelial, urinary and blood serum [21]. However, rat and mouse allergenic protein identification remains difficult in clinical routine. Specific IgE to rat and mouse proteins are not available in clinical routine. Technological advances in chip-based microarrays and molecular allergology have made it possible to simultaneously detect IgE specific for a broad array of allergens in a single test [59]. The microarray assays use natural or recombinant allergen components on the microchip, though some recent assays also include extracts [59]. Microarray tests may be an interesting tool to identify rodent sensitizations and cross-reactivities [48]. However, commercial microarray tests currently detect only few allergenic proteins from rat and mouse. For instance, the ALEX version 2 (ALEX2) (MacroArray Diagnostics, Vienna, Austria) detects sensitization to Mus m 1 and rat epithelium, and the ImmunoCAP Immuno Solid-phase Allergen Chip (ISAC) 112 microarray (ThermoFisher Scientific, Uppsala, Sweden) detects sensitization to Mus m 1.

## Treatment of Laboratory Rat and Mouse Allergy

### Treatment of Symptoms

In the event of an allergy crisis in the LAF, treatment is based on the medical treatment of each of the symptoms: rhinitis, asthma, conjunctivitis, urticaria or anaphylaxis [21, 60]. Regarding laboratory rodent-induced asthma, the impact of biologic therapies on rodent tolerance in the LAF would be interesting to study [61]. Indeed, five biologicals are currently recognized in the treatment of severe asthma: Benralizumab (AstraZeneca), Dupilumab (Sanofi Regeneron), Mepolizumab (GlaxoSmithkline), Omalizumab (Novartis), and Reslizumab (Teva) [62, 63]. No cases of LAW treated with biologicals have been reported so far. Bruton’s tyrosine kinase inhibitors, initially prescribed for B-cell malignancies, are showing promise in the management of allergies. In September 2025, Remibrutinib (Novartis) received United States Food and Drug Administration approval for the oral treatment of chronic spontaneous urticaria in adult patients with symptoms that persist despite treatment with H1 antihistamines [64]. Remibrutinib is also under development for asthma [64, 65].

### Desensitization to Rat and Mouse

No immunotherapy to rat and mouse is commercialized. So far, only one case of immunotherapy in an individual allergic to mice has been reported in the literature [26]. According to Bunyavanich et al., immunotherapy was made with Mus musculus allergenic extract from Greer (0.5 ml epithelium extract in 9.5 ml diluent containing 50% glycerin). A 55-year-old man with an anaphylactic history to mouse underwent a subcutaneous immunotherapy. The immunotherapy protocol started at 0.05 ml of 1:1000 solution and increased weekly over 28 weeks to reach a maintenance dose of 0.50 ml of 1:1 solution. The treatment was well tolerated and efficient. The LAW showed no further symptoms of rhinitis when working with mice after 14 months of immunotherapy. This single case does not allow us to conclude that immunotherapy is effective in treating rat and mouse allergy. However, it highlights the existence of potential curative treatments.

### Prevention

Primary and secondary prevention remains one of the keys to managing an allergy to laboratory rats and mice [56]. Even if a complete eviction is often needed in patients with more severe symptoms, reduction of exposure is efficient in most patients with mild to moderate symptoms [60]. Among the various tasks possible with laboratory rats and mice, some promote greater risk of exposure to allergens. Animal experiments in lab operation rooms give the lowest exposure, whereas cage emptying and cage washing give the highest exposure [66]. Open cages are no longer recommended in LAF because they promote the dispersion of allergens throughout the animal room. Individually ventilated cages (IVC) with positive and negative pressure are preferred to limit the risk of allergen exposure. Use of IVC with positive air pressure largely increase the allergen exposure in the cage washrooms contrary to IVC with negative pressure [66]. Note that, for the well-being of the animal, it is necessary to favor ventilation with an air flow arriving at the level of the cage cover rather than at the level of the animal [67]. Measures limiting exposure to rodent allergens, such as the use of IVC, masks and gloves, do not yet appear to be applied in all LAF [10]. Rat and mouse allergens are very volatile. A recent study show they may be found in the homes of LAW [68]. Cleaning cages and cleaning mouse facilities seem to be the most important work-related factors contributing to higher mouse allergen concentrations at home, along with infrequent changing of bed linen [68]. It is recommended to change the bed linen every 2 weeks minimum in this context [69].

## Conclusion

Despite known preventive measures, the prevalence of rat and mouse allergy among LAW remains stable over the last decade compared to previous data ranging from 4,4% to 30%. The advent of molecular biology, which makes it possible to identify the proteins involved in allergies, allergen immunotherapy and biologic therapy may perhaps lead to the development of personalized treatments in future.

## Key References

- Stave GM, Swift MD, Gochnour MK, Hudson TW, Isakari MT, Behrman AJ. Laboratory Animal Allergy. J Occup Environ Med 2025;67:376–84.
  - The results of this literature review suggest that laboratories have not fully adopted best practices for preventing exposure of laboratory workers to animals such as rats and mice. Workers are therefore still exposed to the risk of allergy.
- Lu J, Zhu H, Yang Q, Xu Y, Huang Z, Sun B. Associations of Protein Classes With Cross-Reactivity and Cross-Sensitization in Furry Animal Allergens: A Component-Resolved Diagnostics Study. J Asthma Allergy 2025;Volume 18:363–75.
  - Findings of this study suggest that serum albumin is the primary factor in cross-sensitization between fur-bearing animals rather than lipocalins.

## Data Availability

No datasets were generated or analysed during the current study.

## Abbreviations

ALEX2: ALEX version 2
CI: Confidence interval
ISAC: ImmunoCAP Immuno Solid-phase Allergen Chip
LAF: Laboratory animal facility
LAW: Laboratory animal workers
PR: Prevalence ratio
SPT: Skin prick tests
IgE: Immunoglobulins E
IVC: Individually ventilated cages
